# Trace the History of HIV and Predict Its Future through Genetic Sequences

**DOI:** 10.3390/tropicalmed7080190

**Published:** 2022-08-17

**Authors:** Zhen Wang, Zhiyuan Zhang, Chen Zhang, Xin Jin, Jianjun Wu, Bin Su, Yuelan Shen, Yuhua Ruan, Hui Xing, Jie Lou

**Affiliations:** 1 Department of Mathematics, Shanghai University, Shanghai 200444, China; 2 Department of Statistics, Columbia University, New York, NY 10027, USA; 3 School of Nursing, University of Rochester, Rochester, NY 14627, USA; 4 Anhui Provincial Center for Disease Control and Prevention, Hefei 230601, China; 5 State Key Laboratory for Infectious Disease Prevention and Control, Collaborative Innovation Center for Diagnosis and Treatment of Infectious Diseases, and National Center for AIDS/STD Control and Prevention, Chinese Center for Disease Control and Prevention, Beijing 102206, China

**Keywords:** HIV/AIDS, Bayesian phylogenetic method, effective reproductive number, primary drug resistance, transmitted drug resistance, dynamic model

## Abstract

Traditional methods of quantifying epidemic spread are based on surveillance data. The most widely used surveillance data are normally incidence data from case reports and hospital records, which are normally susceptible to human error, and sometimes, they even can be seriously error-prone and incomplete when collected during a destructive epidemic. In this manuscript, we introduce a new method to study the spread of infectious disease. We gave an example of how to use this method to predict the virus spreading using the HIV gene sequences data of China. First, we applied Bayesian inference to gene sequences of two main subtypes of the HIV virus to infer the effective reproduction number (${}_{G}R_{e}\left(t\right)$) to trace the history of HIV transmission. Second, a dynamic model was established to forecast the spread of HIV medication resistance in the future and also obtain its effective reproduction number (${}_{M}R_{e}\left(t\right)$). Through fitting the two effective reproduction numbers obtained from the two separate ways above, some crucial parameters for the dynamic model were obtained. Simply raising the treatment rate has no impact on lowering the infection rate, according to the dynamics model research, but would instead increase the rate of medication resistance. The negative relationship between the prevalence of HIV and the survivorship of infected individuals following treatment may be to blame for this. Reducing the MSM population’s number of sexual partners is a more efficient strategy to reduce transmission per the sensitivity analysis.

## 1. Introduction

AIDS (Acquired Immunodeficiency Syndrome) is a disease caused by HIV (Human Immunodeficiency Virus) and has become a disease that seriously threatens human health in the world today. In order to alleviate the AIDS epidemic in China, the Chinese government began to implement the “Four Frees and One Care” HIV/AIDS free treatment policy in 2003 [1,2]. This policy has greatly improved the patient’s survival rate and the patient’s life quality. On the other hand, the high mutation rate of HIV virus has led to the generation of HIV-resistant strains [3]. The spread of drug-resistant strains causes a waste of national medical resources.

We want to know the effect of the free treatment policies, especially the drug resistance situation of HIV/AIDS by dynamic models. It is hard to avoid parameter acquisition when performing prediction with dynamics models. Some parameters are general parameters, which are common to both China and abroad. We can assign these values by searching relative literature. Some might be greatly different due to different countries or different treatment strategies, so they need be fitted by monitoring data. However, such areas happen to lack monitoring data except for some gene sequences of HIV patients being detected before treatment. In this manuscript, we will show how to combine the gene sequences of HIV and the dynamic model to study the dynamics of HIV spreading.

HIV is an RNA virus. RNA viruses have a short reproduction time and a high mutation rate, which can easily lead to a large number of genetic mutations during the transmission process. Therefore, during the spread of HIV, relevant signals will be left in the sampled virus sequence. In recent years, researchers have begun to conduct retrospective studies on the spread of the HIV virus on the genetic level. Most of these studies are based on genetic sequences to build molecular networks of transmission using Bayesian evolution analysis [4,5,6,7,8,9,10,11,12]. For example, in 2008, Lewis et al. used the Bayesian framework to conduct a phylogenetic analysis of 2126 HIV gene sequences in London, and they constructed a network for HIV transmission, revealing that the local HIV/AIDS epidemic can be traced back to the late 1990s [13]. In 2018, Zhao et al. constructed a molecular network of the spread of MSM in Beijing based on the HIV gene sequence, and they proposed the need to strengthen education and intervention for people with potential high risk. These findings have important implications for the parameterization of epidemiological models and the design of intervention strategies.

Traditional dynamic models can be used to predict the spread of virus in populations [14,15,16,17,18,19,20,21,22], and the Bayesian evolution analysis of virus separated from the infected people can trace the historical dynamics of virus transmission [13,23,24]. However, the work connecting the gene sequences and the dynamic model has not yet been seen. This manuscript will use Anhui province as an example, combine genetic sequence molecular evolution analysis and system dynamics models to conduct an in-depth research on the HIV/AIDS epidemic in the content of the HIV-free treatment policy in China, and thus predict the drug resistance spreading in the next few years.

## 2. Research Methodology

### 2.1. Gene Sequence Analysis

To trace the history of transmission dynamics of HIV, we sampled totally 360 HIV-pol gene sequences (CRF 01AE (150), CRF 07BC (190), CRF 08BC (9) and CRF 5501B (11)) from 360 HIV patients who were infected with HIV through sexual behavior in Anhui Province. This data set is sampled between October 2017 and September 2018. Considering that the proportions of the CRF 08BC and CRF 5501B are too small, we only analyzed the subtypes CRF 01AE and CRF 07BC, comprising a total of 340 HIV virus gene sequences. We applied Mega for sequence alignment. The total length of the pol gene that we studied was 1075 bp.

The selection of a nucleotide substitution model is an important step during the phylogenetic analysis. Common nucleotide substitution models include the HKY (+Γ+I) model, GTR (+Γ+I) model, and so on [25]. These different substitution models determined different evolutionary distances and different phylogenetic trees [26]. Here, we used software Phylosuite to select the most suitable substitution model according to the Akaike Information Criterion (AIC) [27], where AIC was defined as: AIC=2k−2ln(L), in which *k* is the number of corresponding parameters and *L* is the likelihood value. Method-of-moments was used to estimate the AIC method. The model with lower AIC value is the more suitable model. After calculating the AIC score, the HKY + Γ + I model and the uncorrelated exponential relaxed molecular clock are determined finally.

#### 2.1.1. Bayesian Inference

The birth death skyline (BDSKY) model in BEAST2 is a random description of population change, which allows the extraction of virus transmission dynamics information from a phylogenetic tree [11]. This model is established on Bayesian inferences, which can estimate the effective reproduction number of HIV/AIDS transmission from gene sequences. The Bayesian approach allows us to continuously update our estimate of a set of parameters, Θ, as data become available.

$$P\left(\Theta |data\right)=\frac{P(data|\Theta )\cdot P(\Theta )}{P(data)}.$$

P(Θ), the prior distribution, represents our prior estimates about the true value of Θ. P(data|Θ) is the likelihood distribution. It is also often written as L(Θ|data), which means the probability of observing the data given Θ. For the method to work, it is necessary to calculate the likelihood distribution for all possible values of Θ. P(data) is the model evidence, and it is the same for all possible hypotheses (values of Θ) being considered. P(Θ|data) is the posterior distribution and represents our updated estimate of the value of Θ given the observed data.

The main objective of Bayesian inference is to calculate the posterior distribution of our parameters using our prior beliefs updated with our likelihood. From the posterior distribution, we can determine the most likely values of Θ given the observed data. Since we are usually only interested in relative probabilities of different hypotheses, P(data) can be left out of the calculation, and we write the model form of Bayes’ theorem as

P(Θ|data)∝P(data|Θ)·P(Θ)

where ∝ means “proportional to”.

#### 2.1.2. The Effective Reproduction Number ${}_{G}R_{e}\left(t\right)$ Infered from Gene Sequences

Since we will study two different subscript gene sequences, we use j=07BC for the subtype CRF 07BC and j=01AE for the subtype CRF 01AE. We will deduce the propagation dynamics of the two subtypes separately. For each subtype *j*, the BDSKY process can conclude three parameters: transmission rate $\lambda_{j}$, removal rate $\gamma_{j}$ and sampling probability $\psi_{j}$. This process allows individuals to “birth” ($\lambda_{j}$) or “death” ($\gamma_{j}$ and $\psi_{j}$) at any point in time. A “birth event” corresponds to an individual’s infection, and a “death event” corresponds to an infected individual becoming non-infected (i.e., removed from the infected compartment because of individual death, successful treatment, or individual behavior change). Then, the effective reproduction number $R_{e}\left(t\right)$ derived from the gene sequences can be defined as [11]

$${}_{G}^{j}R_{e}\left(t\right)=\lambda_{j}\left(t\right)/\left(\mu_{j}\left(t\right)+\psi_{j}\left(t\right)\right),$$

in which the left-hand subscript *G* denotes that the effective reproduction number is derived from the gene sequences. For estimating ${}_{G}^{j}R_{e}\left(t\right)$ of subtype *j*=07BC or 01AE, the Bayes’ theorem that we use is

(1) $$\begin{array}{c} P{(}_{G}^{j}R_{e}\left(t\right)|gene\;sequences)\propto P\left(gene\;sequences{|}_{G}^{j}R_{e}\left(t\right)\right)\cdot P{(}_{G}^{j}R_{e}\left(t\right)). \end{array}$$

In order to catch the characteristics of transmission in different historical stages, we divided the period from the origin of each subtype HIV sequences to the sampling time of the last sample into a total of six segments. Then, from each subtype gene sequence of *j*=07BC and 01AE, we can infer the effective reproduction number ${}_{G}^{j}R_{e}\left(t\right)$ during each segment *t*, t=1,2,…,6 through Equation (1). Hence, ${}_{G}^{j}R_{e}\left(t\right)$ is a piece wise function for each of the two subtypes, which is a constant during the same time segment *t*. It refers to the average number of new infections caused by an infected person at a certain time point *t* during the outbreak. We use the Markov Chain Monte Carlo (MCMC) method to achieve Bayesian phylogeny inference.

### 2.2. Transmission Dynamics Model

#### 2.2.1. A Nested Model and Its Four Submodels

**Figure 1:**
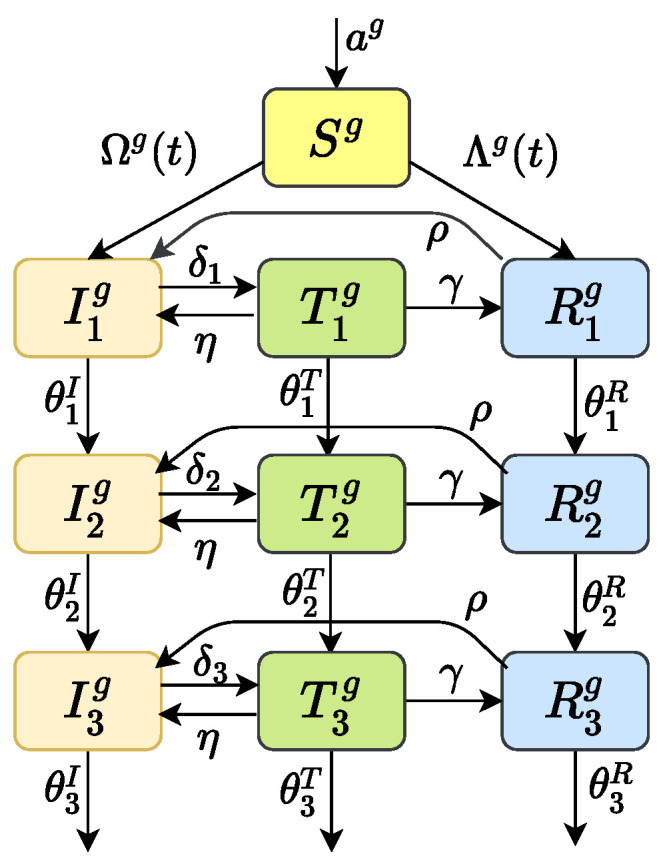
Transmission process diagram. The superscript *g* can be w,mw,mm and *b*, respectively, which represents the four types of subgroup populations. Parameter *a* is an input of susceptible individuals; $\theta_{i},i=1,2,3$ are the disease progression rates in infection stage 1, 2 and 3, respectively; $\delta_{i},i=1,2,3$ are the treatment rates of patients in stage 1, 2 and 3, respectively; η is the percentage of patients who give up HAART; γ is rate of drug resistance after HAART; ρ is the percentage of patients who are no longer resistant.

To predict the prevalence of HIV drug-resistant strains, we considered the transmission dynamics of HIV/AIDS through sexual route, including gay, straight and bisexual. The whole population was divided into four subgroups: heterosexual women who only have sex with men (marked as *w*), heterosexual men who only have sex with women (marked with mw), homosexual men who only have sex with men (marked with mm), and bisexual men who have sex with both women and men (marked as *b*). The population was divided into susceptible people *S*, infected people *I* (infected but not receiving treatment), treated people *T*, and drug-resistant people *R* according to the infection. Considering that the HIV infection process has obvious stage characteristics, according to the level of CD4 cells in the patient’s body, we divide the infected people into three stages: the first infection stage $I_{1}$ (CD4 count ≥500cells/mm${}^{3}$); the second infection stage $I_{2}$ (200cells/mm${}^{3}$< CD4 counts <500cells/mm${}^{3}$); and the third stage of infection $I_{3}$ (AIDS stage, CD4 count ≤200cells/mm${}^{3}$). Similarly, $T_{i}$ and $R_{i}$, i=1,2,3 can be explained. The details of the model can be found in Figure 1. When the superscript *g* in the transmission process diagram Figure 1 is taken as w,mw,mm and *b*, it represents the four types of subgroups. See Appendix A.1 for the corresponding dynamic model.

The standard treatment of HIV/AIDS in China has gone through four periods: the period before 2002 featured no treatment; the period from 2003 to 2011 was when the treatment was started when the CD4 count was less than 200cells/mm${}^{3}$; the period from 2012 to 2015 was when the treatment was started when the CD4 count was less than 500cells/mm${}^{3}$ and the period after 2015 was when the treatment was started soon after discovery. We call these periods I (1992–2002), II (2003–2011), III (2012–2015) and IV (2016–now), respectively.

Due to different prevention and control policies being held in different historical stages, the dynamics of HIV transmission have different stage characteristics. So, our model (Figure 1) in fact is a nested model. For the four different historical treatment periods I, II, III and IV, we can simplify the model to be four submodels by letting some parameters be zero based on its history characters; see Table 1 for the four different submodels. According to the “next-generation operator” method in the literature [28], we can obtain the basic reproduction number ${}_{M}^{k}R_{0}$ for each submodel of corresponding historical period k= I, II, III and IV (see Appendix A.2). Here, the left-hand subscript *M* means that the basic reproduction number ${}_{M}^{k}R_{0}$ was obtained from k= I, II, III or IV, one of the four models respectively.

#### 2.2.2. The Effective Reproduction Number ${}_{M}R_{e}\left(t\right)$ Inferred from the Dynamic Submodels

For a general dynamic model of an infectious disease, the basic reproduction number $R_{0}$ is the expected number of infections directly generated by one case given that all individuals are equally susceptible. As the infection spreads, the susceptibility of the population decreases. The effective reproduction number, $R_{e}\left(t\right)$, is related to the basic reproduction number $R_{0}$, by $R_{e}\left(t\right)=R_{0}\cdot S\left(t\right)/N\left(t\right),$ where S(t)/N(t) is the average susceptibility of the population. $R_{e}\left(t\right)$ is often used as an indicator of the effectiveness of interventions, such as social distancing measures, to contain the spread of a virus. If $R_{e}\left(t\right)$ is greater than 1.0, the infection is growing at an exponential rate. If $R_{e}\left(t\right)$ is at 1.0, the spread is sustained at a linear rate. If $R_{e}\left(t\right)$ is less than 1.0, the infection is spreading at a slower pace and will eventually die out.

Specific to our study on HIV/AIDs spreading during four different historical periods k= I (1992–2002), II (2003–2011), III (2012–2015), and IV (2016–now), let us make

(2) $$\begin{array}{c} {}_{M}^{k}R_{e}\left(t\right){=}_{M}^{k}R_{0}\frac{S^{k}\left(t\right)}{N^{k}\left(t\right)} \end{array}$$

where the basic reproduction number ${}_{M}^{k}R_{0}$ was obtained from Section 2.2.1 and $S^{k}\left(t\right)$ and $N^{k}\left(t\right)$ represent the susceptible population and the total target population of each historical period k= I, II, III and IV, respectively [29].

## 3. Results

### 3.1. Tracing Back the Dynamic History of HIV/AIDS through Bayesian Inference

The MCMC converged after 20 million iterations for both subtypes. We discarded the first 2 million iterations. The phylogenetic tree and model parameters were sampled every 1000 iterations. All parameter estimates showed the effective sample size (ESS) of more than 200. Some results are shown in Table 2.

For each data set and substitution model, we analyze them with an uncorrelated exponential relaxed molecular clock and a strict clock model. In the study of virus evolution, we used nucleotide substitution models including the HKY (+Γ+I) model and GTR (+Γ+I) model [25]. The most appropriate combination was selected according to the Akaike Information Criterion (AIC) [27], where AIC was defined as AIC=2k−2ln(L), in which *k* is the number of corresponding parameters and *L* is the likelihood value. Method-of-moments was used to estimate the AIC method. Finally, the uncorrelated exponential relaxed molecular clock and HKY + Γ+ I model is determined by calculating the AIC value. The important epidemiological parameter estimation and their 95% HPD of subtype CRF 01AE and CRF 07BC are shown in Table 2.

The results in Table 2 show that the most recent common ancestor (MRCA) to the 150 CRF 01AE subtype gene sequences was around 1982, and the 95% highest posterior density (HPD) is between 1973 and 1990. Correspondingly, the MRCA to these 190 CRF 07BC subtype gene sequences was relatively late, around 1992 (95% HPD: 1976-1998). Most importantly, we obtained the effective reproduction number ${}_{G}^{j}R_{e}\left(t\right)$ over time segment *t* from the two subtypes, respectively, in which t=1,2,…,6 represents the time segment. The effective reproduction number ${}_{G}^{j}R_{e}\left(t\right)$ of the two subtypes showed some similar transmission characteristics in terms of time: both of them were in a relatively stable state in the early stage, kept around 1.6 with slight fluctuations, and showed a slight downward trend from 2008 to 2012. However, things changed a lot around 2012: for some reason, the effective reproduction number increased rapidly and then remained at about 2.2.

When we study the history of HIV transmission dynamics in the entire population through the two effective reproduction numbers ${}_{G}^{j}R_{e}\left(t\right),j=01AE,07BC$(t),j=1,2, we will have to integrate them together. The two effective reproduction numbers originated at different times but ended at the same time. We took the common historical stage of them. So, the research period was from 1992 to 2018. During this common historical period, we denote the average of the effective reproduction number for the entire population as ${}_{G}R_{e}\left(t\right)$, which changes along with time *t*.

In order to figure out the formula for ${}_{G}R_{e}\left(t\right)$, we used the proportion of the number of sequences of each subtype in the total gene sequence as its weight in the effective reproduction number, since subtypes widely disseminated in the population should have more contribution for ${}_{G}R_{e}\left(t\right)$. Let $M_{j}$ be the sequence number of gene subtype *j* (j=01AE, 07BC). Then, the weighted average effective reproduction number of the entire population at time *t* can be described by Formula (3).

(3) $$\begin{array}{c} {}_{G}R_{e}\left(t\right)=\frac{\sum_{j}{}_{G}^{j}R_{e}\left(t\right)M_{j}}{\sum_{j}M_{j}},\;\left(j=01AE,07BC;t=1992,1993,\ldots ,2018\right) \end{array}$$

**Figure 2:**
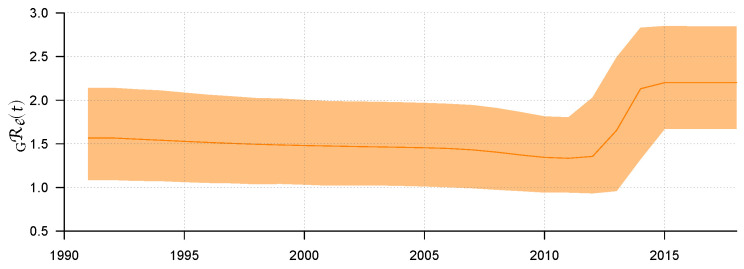
The weighted average of the effective reproduction number ${}_{G}R_{e}\left(t\right)$ and its 95% HPD interval.

Use *R* software to visualize ${}_{G}R_{e}\left(t\right)$; then, the result can be found in Figure 2. Figure 2 shows the median line of ${}_{G}R_{e}\left(t\right)$ and its 95% HPD interval from 1992 to 2018. Before 2011, we can see that the epidemic showed a relatively stable transmission trend through sexual route. The value of ${}_{G}R_{e}\left(t\right)$ remained stable and slightly decreased, with a median value of about 1.33 (95% HPD: 0.94–1.8). However, from 2011 to 2014, ${}_{G}R_{e}\left(t\right)$ rose rapidly to 2.13 (95% HPD: 1.32–2.83) for some reason. After 2015, ${}_{G}R_{e}\left(t\right)$ remained stable, with its median value maintained at approximately 2.20 (95% HPD: 1.67–2.85). Some explanations will be given later on the possibility of ${}_{G}R_{e}\left(t\right)$ rising in this period.

### 3.2. Dynamic Model Analysis: Parameter Estimations and Predicting the Transmission of HIV/AIDS

#### 3.2.1. Parameter Estimations

As we all know, an important step in the prediction of infectious disease spreading is to obtain (or estimate from data) parameters values. For most of the general parameters we treated them as constants from references. Details of the values and references can be found in Appendix A.4. However, there are still some epidemiological related parameters that we are not sure of and need to be estimated from data.

Unlike novel coronavirus patients, HIV-infected people do not have obvious infection symptoms. Many HIV infected people do not know that they have been infected for a long time. This prevents the government from obtaining information about people living with HIV as early as possible. Moreover, the Chinese government’s screening efforts for HIV-infected people are far less than those for infected novel coronavirus. As a result, recorded HIV infection data, if available, may not be very accurate and may not be suitable for parameter fitting. In Anhui province, which we studied, there is not much surveillance data on HIV/AIDS that can be used. One form of reliable data available are 340 genetic sequences of HIV-infected people taken before treatment.

**Figure A1:**
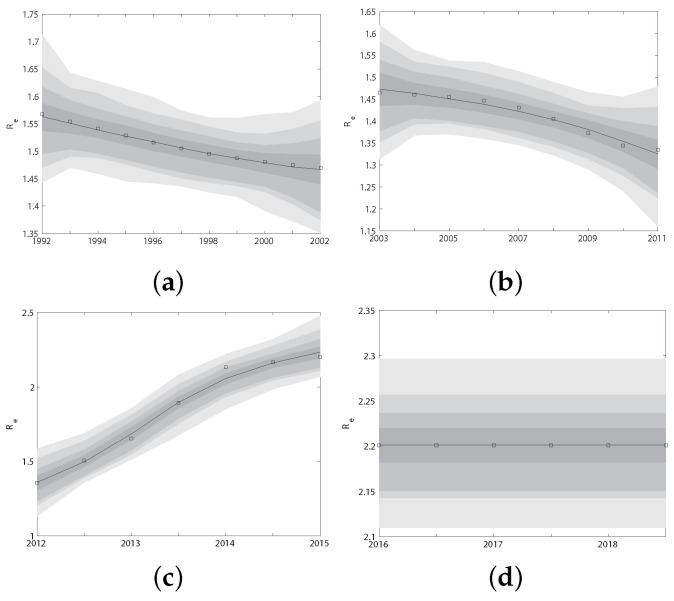
The fitted curves of the two effective reproduction numbers ${}_{G}R_{e}\left(t\right)$ and ${}_{M}R_{e}\left(t\right)$ for four historical periods I (**a**), II (**b**), III (**c**) and IV (**d**), respectively.

In the previous subsections, we have extrapolated the dynamics of HIV transmission (${}_{G}R_{e}\left(t\right)$) from 1992 to 2018 using the 340 genetic sequences. In order to use these gene data to estimate unknown parameters of the model (Figure 1, which consists of four different submodels I (1992–2002), II (2003–2011), III (2012–2015) and IV (2016–now), depending on the historical period from 2003–now), we calculated the effective reproduction number ${}_{M}^{k}R_{e}\left(t\right)$ for each submodel k= I, II, III and IV by Equation (2). Obviously, ${}_{M}^{k}R_{e}\left(t\right)$ is functions of parameters and time *t*. Since the two effect reproduction numbers obtained through different methods should be the same at the same time *t*, i.e., ${}_{M}R_{e}\left(t\right){=}_{G}R_{e}\left(t\right)$, we fit them at the same historical period. In this way, we explored the humanistic and statistical features in different historical stages. We used MCMC to realize this. The algorithm ran for 2.00 million iterations, and we adapted the proposal distribution after 2 million iterations using Geweke’s method [30] to assess convergence. The fitted curves of the two effective reproduction numbers for four historical periods I, II, III and IV are shown in Figure A1 in Appendix A.4. The fitted parameters obtained from the above four periods are shown in Table 3. The value of these parameters will be referred to as baseline parameter values in the following research.

#### 3.2.2. HIV/AIDS Epidemic Trend under Baseline Parameters

**Figure 3:**
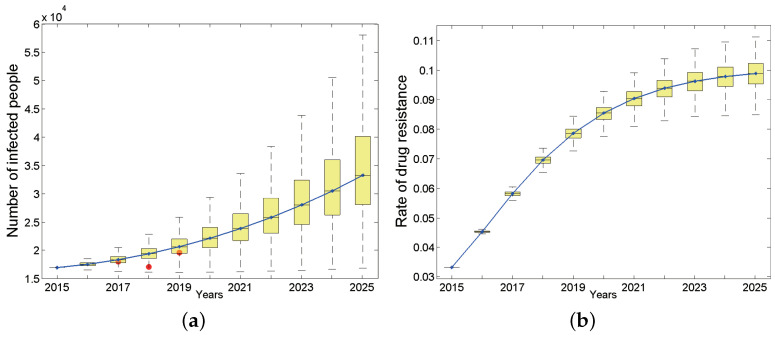
The number of HIV-positive people in the entire population (**a**) and drug resistance rate (**b**), where the continuous curve is the median line. Red dots in (**a**) are reported cases of 2017, 2018 and 2019 respectively.

We first studied the dynamic process of the total number of infected people and the proportion carrying drug-resistant strains over time from 2015 to 2025 in the entire population of Anhui province under baseline parameters (Figure 3). The results show that the total number of HIV/AIDS infections during this period appears to be an upward trend, and the total number of surviving infections will increase to 33,260 by 2025 (95% confidence interval (CI): 28,096–40,090, Figure 3a). The number of new infections is also increasing and will reach 4522 (95% CI: 3366–6130) by 2025. According to the Anhui Provincial Health and Family Planning Commission, from 2017 to 2019, the cumulative reports of HIV/AIDS surviving patients whose current address is Anhui province are 18619 [31], 17183 [32] and 19604 [33], respectively. They are indicated by red dots in Figure 3a. Considering that the detection rate of HIV infections in Anhui province has not reached 100%, the number of reported cases should be less than the actual number of HIV infections. Based on the reported HIV-positive people and the HIV testing rate [31] in the corresponding year, we have reason to say that our predicted results are basically consistent with the actual situation.

The target population of our study is the general population. We studied the proportion of each population in the total new infections under baseline parameters from 2015 to 2025. The results showed that the proportion of heterosexual HIV-positive people decreased from 30.28% in 2015 to 20.43% in 2025 (95% CI: 15.92–26.09%, of which female infected people accounted for 15.61%, while heterosexual males accounted for 4.82%). In contrast, the proportion of HIV-positive MSM (homosexual and bisexual men) has steadily increased. By 2025, 78.84% (95% CI: 68.97–88.70%) of new HIV infections will be infected through homosexual sex behavior. Among them, homosexual HIV-positive people accounted for 53.87%, and bisexual HIV-positive people accounted for 24.97%.

Regarding the subpopulations, although the HIV infection rate among heterosexual men has declined and tends to be stable, the rates in other populations have shown steady upward trends. By 2025, the HIV-positive rate among heterosexual women will reach 0.25% (95% CI: 0.21–0.28%), while the HIV-positive rate among heterosexual men will be lower, only about half of the positive rate of women (0.12%, 95% CI: 0.10–0.13%). In contrast, men who have sex with men belong to high-risk groups, and their HIV positive rate is dozens of times that of heterosexual men: the HIV infection rate of bisexual men is 2.44% (95% CI: 1.88–3.12%), while that of homosexual men is much higher, reaching 3.62% (95% CI: 2.81–4.64%) by 2025.

Considering that large-scale treatment for HIV-infected people in China has lasted for quite a long time, we are concerned that drug-resistant strains (secondary drug resistance) will appear in the patients due to poor medication compliance, which will lead to the wide spread of drug-resistant strains (primary drug resistance) in the population. Unsurprisingly, the proportion of HIV-positive people carrying drug-resistant strains in Anhui province has shown a rapid upward trend (Figure 3b) and will basically reach the highest point of 9.88% by 2025, which corresponds to 3282 (95% CI: 2862–3812) in terms of quantity. The proportion of primary drug-resistant patients in the total newly infected population will also increase over time. By 2025, the number of primary drug-resistant patients will account for 9.63% of total patients carrying drug-resistant strains (95% CI: 6.56–14.2%). In addition, the proportion of primary drug-resistant patients among total new infections first showed an upward trend, then tended to be stable each year, and finally will increase to 7.01% (95% CI: 6.61–7.44%) by 2025.

#### 3.2.3. Influence of Intervention Measures on HIV/AIDS Epidemic Trend

In order to implement the “Healthy China 2030” program and deepen the medical and health system reformation, China’s “13th Five-Year Plan for Combating and Prevention of AIDS” in 2017 set the “90–90–90” goal. That is, the proportion of infected persons detected by testing should be above 90%; the proportion of diagnosed infected persons receiving antiviral treatment should be above 90%; the treatment success rate of infected persons receiving antiviral treatment should be above 90% [34] by 2020. In the baseline parameters, the treatment rates of the three different infection stages were 50.19% (95% CI: 21.55%, 78.83%), 75.28% (95% CI: 60.86%, 89.71%) and 74.94% (95% CI: 60.57%, 89.30%). These treatment rates are still far from the second “90%”. Next, we consider what will happen if the treatment rate increases to the target state in 2020.

**Figure 4:**
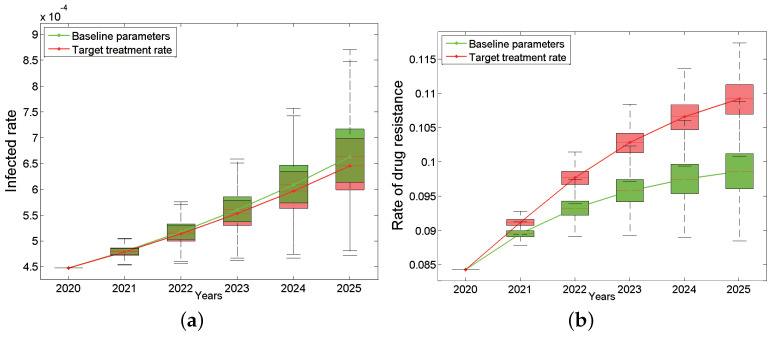
Impact of target treatment rates on the HIV/AIDS epidemic: the infected rate (**a**) and the rate of drug resistance (**b**). The green box plots are based on the baseline parameters, while the red ones are based on target treatment rate. The continuous green and red curves are their median lines respectively.

The total HIV infection rate and drug resistance of Anhui province from 2020 to 2025 under the “90%” treatment rate are shown in Figure 4, which is a comparison chart between the baseline and the target situation. The results show that the total infection rate still keeps increasing (the green curve in Figure 4a) even under the “90%” treatment rate, which is only slightly lower than the baseline parameters (the red curve in Figure 4a). Even more surprising, the total drug resistance rate after intensified treatment will increase more rapidly than the baseline situation, reaching 10.92% (95% CI: 10.69–11.12%) by 2025, which is seen as the red curve in Figure 4b. In addition, the proportion of primary drug resistance in new infections has also shown an increasing trend compared with baseline parameters.

In short, when we increase the treatment rate to 90%, the total infection rate can be reduced only by 0.017% by 2025, but in turn, the total drug resistance rate and the proportion of primary drug resistance in new infections will increase 1.06% and 1.12%, respectively. What prevents ART treatment from playing an effective role in the epidemic? Is the current drug treatment effect not good enough? If the new first-line drugs can improve the treatment effect (including reducing the infectivity and prolonging the lifespan), how much will the epidemic be improved?

**Figure 5:**
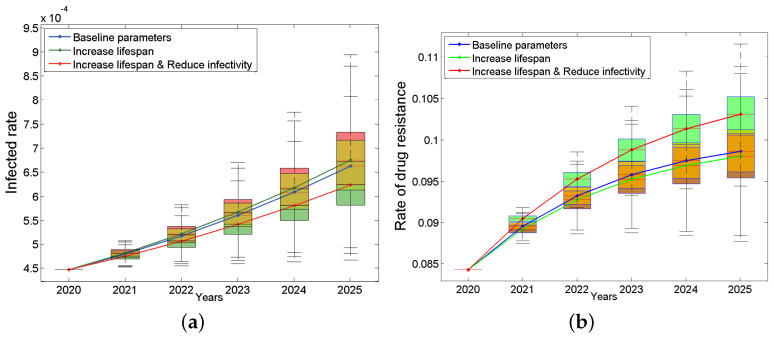
Impacts of improving the treatment effect on the infected rate (**a**) and the rate of drug resistance (**b**). The blue box plots are based on the baseline parameters, the green ones are based on increase lifespan, and red ones are based on both increase lifespan and reduce infectivity. The continuous blue, green and red curves are their median lines respectively.

To answer these questions, we assume the following two situations: (a) the new drug can only prolong the lifespan after treatment by 20% (it can survive for 24 years after extension); (b) the new drug can not only prolong the life after treatment by 20% but also reduce the infection rate by 20%. The results are shown in Figure 5. We were surprised to find that assumption (a) will make the epidemic worse instead (Figure 5a, green line). This may be because patients will have more risky sexual behaviors due to longer lifespan. However, assumption (b) will slow down the trend (Figure 5a, red line). In terms of drug resistance, the drug resistance rate will decrease slightly under assumption (a) (Figure 5b, green line) and increase under assumption (b) (Figure 5b, red line). The main reason may be that treatment only suppresses the non-resistant strains, which may instead give drug-resistant strains more space for transmission. This result suggests that prolonging the lifespan of the patients is a double-edged sword. When developing new drugs, we should not only consider how to prolong the life span of patients but also consider how to reduce the infectivity. This conclusion will continue to be discussed in the sensitivity analysis.

#### 3.2.4. Sensitivity Analysis

In previous studies, we unexpectedly found that merely increasing the treatment rate to the target rate (90% treatment rate) could not lead to a significant improvement in HIV epidemic (Figure 5a). In order to find the reason, we did some sensitivity analysis. We find that only when shortening each infectious stage’s life span to less than 25% of its baseline values can the epidemic be controlled. Details can be found in Appendix A.5. In the process of new drug development, if we cannot effectively reduce the infection rate while continuing to pursue prolonging the life-span of patients after treatment, the epidemic will keep rising as a consequence. We speculate that this may be one of the reasons that the effective reproduction $R_{e}^{g}$ has been increasing since 2012 (Figure 2). In other words, it is difficult to control the epidemic only by developing new drugs in a short period. So, what are the key factors to control the HIV/AIDS epidemic?

To answer this question, we conducted a sensitivity analysis on the number of sexual partners in different populations. An interesting fact is that changing the number of heterosexual people’s sexual partners has little impact on the epidemic, and it almost can be negligible while comparing with the two subpopulations of homosexual sexual behavior who contributed a significant effect to the epidemic. As long as the number of bisexual men’s annual sex partners is controlled below 6, and meanwhile, the number of homosexual men’s annual sex partners is controlled below 4, the epidemic can be controlled. Details can be found in Appendix A.6.

## 4. Discussion and Conclusions

Generally speaking, predictive models for infectious diseases generally cannot avoid the problem of parameter estimation. After all, not all parameter values are easy to obtain. Even if some foreign literature mentions the value range of certain parameters, there may still exist differences due to different races. To some degree, parameter values determine the credibility of predictions. Facing the complex history of HIV treatment in China, different historical stages may have different values even for the same parameter. We innovatively carried out data mining on virus gene sequence detected from the infected people, so as to fit some parameter values which are difficult to obtain by social surveys or laboratory tests, making our prediction results more reliable. However, other non-Bayesian research methods are also good choices for the mathematical modeling of infectious diseases with a large number of reported data [35].

The idea of combining gene sequences with dynamic models came from the absence of monitoring data when doing model fitting. Unlike novel coronavirus patients, HIV-infected people do not have obvious infection symptoms. Many HIV-infected people do not know that they have been infected for a long time. This prevents the government from obtaining information about people living with HIV as early as possible. Moreover, the Chinese government’s screening efforts for HIV-infected people are far less than those for individuals infected with novel coronavirus. As a result, recorded HIV infection data, if available, may not be very accurate and may not be suitable for parameter fitting. In Anhui province, which we studied, there is not much surveillance data on HIV/AIDS that can be used. The only reliable data available for use are 340 genetic sequences of HIV-infected people taken before treatment. HIV is an RNA virus. This gives it the ability to mutate faster, and these mutations show up in the entire genetic sequence of the virus from generation to generation. In other words, the evolutionary history of HIV is hidden in its genetic sequences. Since both gene sequences and dynamic models can describe the transmission dynamics of viruses, it should be possible to combine them for the purpose of learning from each other. This is our motivation to finish this manuscript.

What is more, most dynamic models generally only consider heterosexuality and homosexuality when classifying the target population, and they will not further subdivide homosexual people. Our study divided the MSM population into homosexual men and bisexual men to analyze the importance of bisexual men in the spread of HIV/AIDS. These bisexual men may be biological and psychological bisexuals; also, it may be that homosexuals are forced to marry women under the pressure of public opinion to cover up their particular sexual orientation. We generally think that such people are a bridge that links HIV/AIDS from high-risk groups to the general population. Thus, cutting off this bridge will slow down the HIV epidemic. However, the result of our model shows an opposite result: if bisexual men with the unchanged number of sexual partners disconnect from men, the epidemic will slow down a lot; otherwise, it will lead to an increase in the epidemic. For more details, please read Appendix A.7.

In “China’s 13th Five-Year Plan for Containment and Prevention of AIDS”, China advocates that HIV/AIDS prevention and treatment should achieve three 90%: the proportion of infected people and patients who have been diagnosed and know about their infection status should reach 90% or more; the proportion of infected people and patients who meet the treatment conditions receiving antiviral treatment should be more than 90%; the success treatment rate of infected people and patients receiving antiviral treatment should be more than 90%. These three 90% involve screening, treatment and drug development. According to our findings, if no more effective drugs are available, simply increasing the treatment rate will cause a slight decrease in the number of new infections and the number of people with primary drug resistance. Moreover, the epidemic is still on the rise; that is to say, the increase of treatment rate cannot effectively control the epidemic, and the prevention and control effect is not ideal. A similar phenomenon was also found in the study [36] by Lou Jie et al. A plausible explanation is that after treatment, the life expectancy of HIV-infected patients is extended, but the current drug’s effect is not so good at reducing the infection rate, thus creating a greater possibility of transmission instead. In the meantime, free treatments of HIV have not yet covered the entire course of patients, so once drug resistance develops, the treatment led to failure.

Therefore, after the implementation of the “90–90–90” strategic measures, continuous improvements of the treatment rate did not bring the HIV epidemic under control but inversely caused more drug resistance. The Chinese government needs to intervene in various aspects of different groups of people in combination with other perspectives in order to curb the current domestic AIDS epidemic. The sensitivity analysis on the two critical parameters, infection rate after treatment and progress rate of disease, indicates neither one is the most critical factor to impact the epidemic. It would be easier to control the epidemic by reducing the number of sexual partners of the two subpopulations in the MSM group through publicity and other means. In addition, it is worth mentioning that considering the absolute leading role of homosexual people in the epidemic at the present stage, we should tilt most of the resources to this group so that we can benefit from spending money wisely.

In short, gene sequences can tell us the history of HIV/AIDS spreading and the dynamic model can tell us its future. Combining the two together is an innovative approach, especially for epidemics where reliable surveillance data are lacking. In addition, we believe that this new method is not only suitable for HIV but also for other RNA viruses, such as novel coronavirus. Of course, our method is not perfect, since the influencing factors considered by the Bayesian model are relatively simple. However, at the very least, genetic sequence data can be used as a supplement to macro surveillance data for epidemic prediction.

## Figures and Tables

**Table 1:** Submodels of different historical periods and corresponding assumptions.

Submodel	Assumptions	Historical Period
I	$\delta_{i}=0$ and $T_{i}^{g}=0,R_{i}^{g}=0$, i=1,2,3, g=w,mw,mm,b	1992–2002
II	$\delta_{i}=0$ and $T_{i}^{g}=0$, i=1,2, g=w,mw,mm,b	2003–2011
III	$\delta_{1}=0$ and $T_{1}^{g}=0$, g=w,mw,mm,b	2012–2015
IV	The full model	2016–now

**Table 2:** Epidemiological parameter estimations and their 95% HPD of subtype CRF 01AE and CRF 07BC.

Subtype		MRCA	The Effective Reproduction Number j=01AE,07BC					
		**Median**	${}_{G}^{j}R_{e}\left(1\right)$	${}_{G}^{j}R_{e}\left(2\right)$	${}_{G}^{j}R_{e}\left(3\right)$	${}_{G}^{j}R_{e}\left(4\right)$	${}_{G}^{j}R_{e}\left(5\right)$	${}_{G}^{j}R_{e}\left(6\right)$
CFR01AE	95% HPD lower 95% HPD upper	1982	1.64	1.47	1.42	1.37	1.23	2.17
		1973	1.08	0.97	0.94	0.91	0.83	1.61
		1990	2.30	2.08	2.00	1.94	1.72	2.87
CFR07BC	95% HPD lower 95% HPD upper	1992	1.58	1.52	1.51	1.50	1.40	2.21
		1976	1.15	1.11	1.10	1.10	1.03	1.72
		1998	2.10	2.00	1.98	1.97	1.81	2.75

**Table 3:** Model parameters and the 95% HPD fitted by the two effective reproduction numbers.

Parameters	Description	Value	Fitted from Submodel
$a^{w}$	input of heterosexual women	343,780	IV
$a^{mw}$	input of heterosexual men	358,200	IV
$a^{mm}$	input of homosexual men	4974 (2936, 7012)	IV
$a^{b}$	input of bisexual men	4646 (2662, 6630)	IV
$c^{w}$	the average number of sexual partners per year for heterosexual women	1.9957 (0.846, 3.1454)	IV
$c^{mw}$	the average number of sexual partners per year for heterosexual men	2.0946 (0.8514, 3.1742)	IV
$c^{mm}$	the average number of sexual partners per year for homosexual men	5.4984 (2.0701, 7.8565)	IV
$c^{b}$	the average number of sexual partners per year for bisexual men	5.5765 (2.1816, 8.3334)	IV
ϵ	transmissibility ratio of drug-resistant to untreated wild strain carriers	0.43983	II
$1/\theta_{1}^{T}$	course of the disease after treatment in stage 1	7.4030 (6.8823, 8.0000 )	IV
$1/\theta_{2}^{T}$	course of the disease after treatment in stage 2	10.8948 (10.0000, 12.5000)	III
$1/\theta_{3}^{T}$	course of the disease after treatment in stage 3	4.1969(3.3333, 4.7619 )	II
$\delta_{1}$	treatment rate of stage 1	0.50188 (0.2155, 0.7883)	IV
$\delta_{2}$	treatment rate of stage 2	0.75282 (0.6086, 0.8971)	IV
$\delta_{3}$	treatment rate of stage 3	0.74938 (0.6057, 0.8930)	IV
*v*	the percentage of male partners of bisexual men	0.62567 (0.4549, 0.7965)	IV

**Figure A2:**
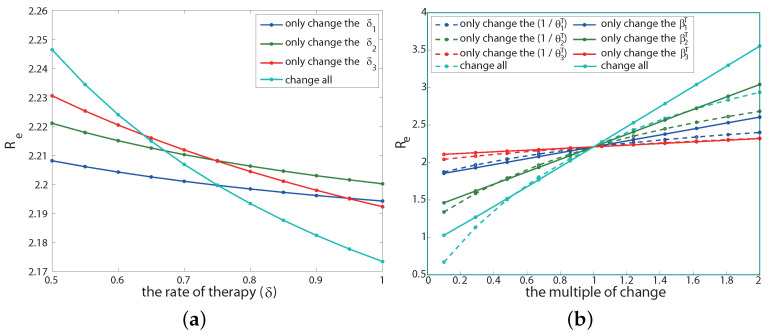
Sensitivity analysis of treatment rate (**a**), infection rate after treatment (solid line in (**b**)) and course of the disease after treatment (dotted line in (**b**)) to $R_{0}^{d}\left(IV\right)$.

**Figure A3:**
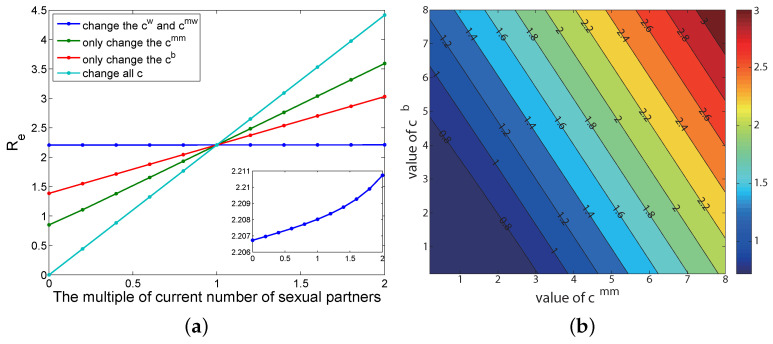
Sensitivity analysis of sexual partners to the reproduction number. (**a**) Only the number of annual sexual partners of various groups of people is changed and other parameters remain unchanged. (**b**) The impact of the number of sexual partners of bisexual men and the number of heterosexual men on the reproduction number.

**Figure A4:**
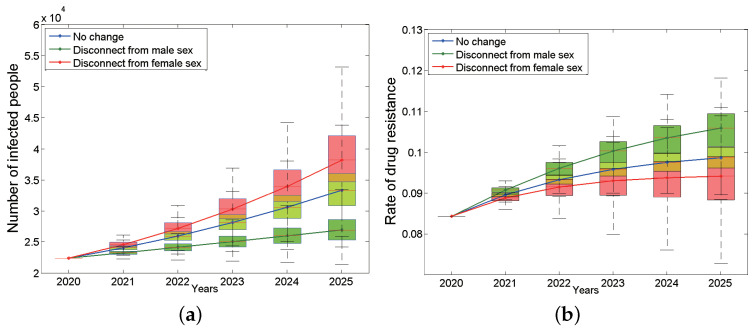
The impact of changing the sexual preferences of bisexual men on the on the number of infected people (**a**) and the rate of drug resistance (**b**). The blue box plots are the situation of no change, the green ones are the situation that disconnect from male sex, and the red ones are the situation that disconnect from female sex. The continuous blue, green and red curves are their median lines respectively.

## Data Availability

All relevant data are within the paper. Additional data cannot be shared publicly based on regulations of the National Center for AIDS/STD Control and Prevention (NCAIDS) of the China Center for Disease Control and Prevention (China CDC). These data are available from the the Institutional Review Boards of the NCAIDS for researchers who meet the criteria for access to confidential data (Tel.: 58900922, Fax: 58900920, E-mail: office606@chinaaids.cn).

## Appendix A

### Appendix A.1. The Dynamic model

(A1) $$\left\{\begin{array}{cc} {\dot{S}}^{g} & =a^{g}-\mu S^{g}-\Omega^{g}\left(t\right)-\Lambda^{g}\left(t\right)\\ {\dot{I}}_{1}^{g} & =\Omega^{g}\left(t\right)-\left(\theta_{1}^{I}+\delta_{1}+\mu \right)I_{1}^{g}+\eta T_{1}^{g}+\rho R_{1}^{g}\\ {\dot{I}}_{2}^{g} & =\theta_{1}^{I}I_{1}^{g}-\left(\theta_{2}^{I}+\delta_{2}+\mu \right)I_{2}^{g}+\eta T_{2}^{g}+\rho R_{2}^{g}\\ {\dot{I}}_{3}^{g} & =\theta_{2}^{I}I_{2}^{g}-\left(\theta_{3}^{I}+\delta_{3}+\mu \right)I_{3}^{g}+\eta T_{3}^{g}+\rho R_{3}^{g}\\ {\dot{T}}_{1}^{g} & =\delta_{1}I_{1}^{g}-\left(\eta +\theta_{1}^{T}+\gamma +\mu \right)T_{1}^{g}\\ {\dot{T}}_{2}^{g} & =\delta_{2}I_{2}^{g}+\theta_{1}^{T}T_{1}^{g}-\left(\eta +\theta_{2}^{T}+\gamma +\mu \right)T_{2}^{g}\\ {\dot{T}}_{3}^{g} & =\delta_{3}I_{3}^{g}+\theta_{2}^{T}T_{2}^{g}-\left(\eta +\theta_{3}^{T}+\gamma +\mu \right)T_{3}^{g}\\ {\dot{R}}_{1}^{g} & =\Lambda^{g}\left(t\right)+\gamma T_{1}^{g}-\left(\theta_{1}^{R}+\rho +\mu \right)R_{1}^{g}\\ {\dot{R}}_{2}^{g} & =\theta_{1}^{R}R_{1}^{g}+\gamma T_{2}^{g}-\left(\theta_{2}^{R}+\rho +\mu \right)R_{2}^{g}\\ {\dot{R}}_{3}^{g} & =\theta_{2}^{R}R_{2}^{g}+\gamma T_{3}^{g}-\left(\theta_{3}^{R}+\rho +\mu \right)R_{3}^{g} \end{array}\right.$$

where $\Omega^{g}\left(t\right)$ and $\Lambda^{g}\left(t\right)\left(g=w,mw,mm,b\right)$ denote the number of newly infected people at the time of *t* at subgroup *g* infected with wild HIV strains and resistant HIV strains, respectively.

(A2) $$\left\{\begin{array}{c} \Omega^{w}\left(t\right)=\frac{c^{w}S^{w}\sum_{i=1}^{3}\left[\beta_{i}^{I}\left(I_{i}^{mw}+I_{i}^{b}\right)+\beta_{i}^{T}\left(T_{i}^{mw}+T_{i}^{b}\right)\right]}{N^{mw}+N^{b}}\\ \Omega^{mw}\left(t\right)=\frac{c^{mw}S^{mw}k\sum_{i=1}^{3}\left(\beta_{i}^{I}I_{i}^{w}+\beta_{i}^{T}T_{i}^{w}\right)}{N^{w}}\\ \Omega^{mm}\left(t\right)=\frac{c^{mm}S^{mm}\sum_{i=1}^{3}\left[\beta_{i}^{I}\left(I_{i}^{mm}+I_{i}^{b}\right)+\beta_{i}^{T}\left(T_{i}^{mm}+T_{i}^{b}\right)\right]}{N^{mm}+N^{b}}\\ \Omega^{b}\left(t\right)=\frac{vc^{b}S^{b}\sum_{i=1}^{3}\left[\beta_{i}^{I}\left(I_{i}^{mm}+I_{i}^{b}\right)+\beta_{i}^{T}\left(T_{i}^{mm}+T_{i}^{b}\right)\right]}{N^{mm}+N^{b}}+\frac{\left(1-v\right)c^{b}S^{b}k\sum_{i=1}^{3}\left(\beta_{i}^{I}I_{i}^{w}+\beta_{i}^{T}T_{i}^{b}\right)}{N^{w}}\\ \Lambda^{w}\left(t\right)=\frac{c^{w}S^{w}\sum_{i=1}^{3}\epsilon \beta_{i}^{I}\left(R_{i}^{mw}+R_{i}^{b}\right)}{N^{mw}+N^{b}}\\ \Lambda^{mw}\left(t\right)=\frac{c^{mw}S^{mw}k\sum_{i=1}^{3}\epsilon \beta_{i}^{I}R_{i}^{w}}{N^{w}}\\ \Lambda^{mm}\left(t\right)=\frac{c^{mm}S^{mm}\sum_{i=1}^{3}\epsilon \beta_{i}^{I}\left(R_{i}^{mm}+R_{i}^{b}\right)}{N^{mm}+N^{b}}\\ \Lambda^{b}\left(t\right)=\frac{vc^{b}S^{b}\sum_{i=1}^{3}\epsilon \beta_{i}^{I}\left(R_{i}^{mm}+R_{i}^{b}\right)}{N^{mm}+N^{b}}+\frac{\left(1-v\right)c^{b}S^{b}k\sum_{i=1}^{3}\epsilon \beta_{i}^{I}R_{i}^{w}}{N^{w}} \end{array}\right.$$

and

(A3) $$\left\{\begin{array}{c} N^{w}=S^{w}+I_{1}^{w}+I_{2}^{w}+I_{3}^{w}+T_{1}^{w}+T_{2}^{w}+T_{3}^{w}+R_{1}^{w}+R_{2}^{w}+R_{2}^{w}\\ N^{mw}=S^{mw}+I_{1}^{mw}+I_{2}^{mw}+I_{3}^{mw}+T_{1}^{mw}+T_{2}^{mw}+T_{3}^{mw}+R_{1}^{mw}+R_{2}^{mw}+R_{2}^{mw}\\ N^{mm}=S^{mm}+I_{1}^{mm}+I_{2}^{mm}+I_{3}^{mm}+T_{1}^{mm}+T_{2}^{mm}+T_{3}^{mm}+R_{1}^{mm}+R_{2}^{mm}+R_{2}^{mm}\\ N^{b}=S^{b}+I_{1}^{b}+I_{2}^{b}+I_{3}^{b}+T_{1}^{b}+T_{2}^{b}+T_{3}^{b}+R_{1}^{b}+R_{2}^{b}+R_{2}^{b}. \end{array}\right.$$

### Appendix A.2. The Basic Reproduction Number $R_{0}^{d}$

Here, we only show how to calculate the most complex basic reproduction number in the IV period, $R_{0}^{d}\left(IV\right)$. The $R_{0}^{d}$ of the other stages (I,II,III) can be simplified from $R_{0}^{d}\left(IV\right)$.

We use the “regeneration matrix” method to find the basic reproduction number $R_{0}^{d}\left(IV\right)$ of the model (A1); the process is as follows:

Step 1:

Construct matrices $f_{0}$ and $v_{0}$, where $f_{0}$ is a column vector of n×1, representing a new infection in each infection state, and $v_{0}$ is a column vector of n×1, representing that each infected state has been transported (not newly infected), where *n* is the number of infected states.

(A4) $$f_{0}^{g}={\left(\Omega^{g},0,0,0,0,0,\Lambda^{g},0,0\right)}^{\prime }$$

then

(A5) $$f_{0}=\left(\begin{array}{c} f_{0}^{w}\\ f_{0}^{mw}\\ f_{0}^{mm}\\ f_{0}^{b} \end{array}\right)$$

(A6) $$v_{0}^{g}=\left(\begin{array}{c} -\left(\theta_{1}^{I}+\delta_{1}+\mu \right)*I_{1}^{g}+\eta *T_{1}^{g}+\rho *R_{1}^{g}\\ \theta_{1}^{I}*I_{1}^{g}-\left(\theta_{2}^{I}+\delta_{2}+\mu \right)*I_{2}^{g}+\eta *T_{2}^{g}+\rho *R_{2}^{g}\\ \theta_{2}^{I}*I_{2}^{g}-\left(\theta_{3}^{I}+\delta_{3}+\mu \right)*I_{3}^{g}+\eta *T_{3}^{g}+\rho *R_{3}^{g}\\ \delta_{1}*I_{1}^{g}-\left(\eta +\theta_{1}^{T}+\gamma +\mu \right)*T_{1}^{g}\\ \delta_{2}*I_{2}^{g}+\theta_{1}^{T}*T_{1}^{g}-\left(\eta +\theta_{2}^{T}+\gamma +\mu \right)*T_{2}^{g}\\ \delta_{3}*I_{3}^{g}+\theta_{2}^{T}*T_{2}^{g}-\left(\eta +\theta_{3}^{T}+\gamma +\mu \right)*T_{3}^{g}\\ \gamma *T_{1}^{g}-\left(\theta_{1}^{R}+\rho +\mu \right)*R_{1}^{g}\\ \theta_{1}^{R}*R_{1}^{g}+\gamma *T_{2}^{g}-\left(\theta_{2}^{R}+\rho +\mu \right)*R_{2}^{g}\\ \theta_{2}^{R}*R_{2}^{g}+\gamma *T_{3}^{g}-\left(\theta_{3}^{R}+\rho +\mu \right)*R_{3}^{g} \end{array}\right)$$

then

(A7) $$v_{0}=\left(\begin{array}{c} v_{0}^{w}\\ v_{0}^{mw}\\ v_{0}^{mm}\\ v_{0}^{b} \end{array}\right)$$

Step 2:

Construct the matrix *F* and *V*, and calculate the value of the Jacobian matrix of $f_{0}$ and $v_{0}$ at the disease-free equilibrium, respectively.

We can easily find the disease-free equilibrium of the model (A1) (disease-free equilibrium) $E_{0}$ is

(A8) $$\left(\frac{a^{w}}{\mu },0,0,0,0,0,0,0,0,0,\frac{a^{mw}}{\mu },0,0,0,0,0,0,0,0,0,\frac{a^{mm}}{\mu },0,0,0,0,0,0,0,0,0,\frac{a^{b}}{\mu },0,0,0,0,0,0,0,0,0\right)$$

The matrix *F*, *V* is the Jacobian matrix of *f*, *v*. Now, the matrix *F*, *V* is represented by a block matrix of 9×9. So, we can obtain $F_{E_{0}}$, $V_{E_{0}}$ as follows.

$$F_{E_{0}}=\left(\begin{array}{c} \begin{array}{cccc} 0 & F_{12} & 0 & F_{14}\\ F_{21} & 0 & 0 & 0\\ F_{31} & 0 & F_{33} & F_{34}\\ F_{41} & 0 & F_{43} & F_{44} \end{array} \end{array}\right)$$

$$V_{0}=\left(\begin{array}{c} \begin{array}{ccccccccc} -\delta^{1}-\mu -\theta_{1}^{I} & 0 & 0 & \eta & 0 & 0 & \rho & 0 & 0\\ \theta_{1}^{I} & -\delta_{2}-\mu -\theta_{2}^{I} & 0 & 0 & \eta & 0 & 0 & \rho & 0\\ 0 & \theta_{2}^{I} & -\delta_{3}-\mu -\theta_{3}^{I} & 0 & 0 & \eta & 0 & 0 & \rho \\ \delta_{1} & 0 & 0 & -\eta -\gamma -\mu -\theta_{1}^{T} & 0 & 0 & 0 & 0 & 0\\ 0 & \delta_{2} & 0 & \theta_{1}^{T} & -\eta -\gamma -\mu -\theta_{2}^{T} & 0 & 0 & 0 & 0\\ 0 & 0 & \delta_{3} & 0 & \theta_{2}^{T} & -\eta -\gamma -\mu -\theta_{3}^{T} & 0 & 0 & 0\\ 0 & 0 & 0 & \gamma & 0 & 0 & -\mu -\rho -\theta_{1}^{R} & 0 & 0\\ 0 & 0 & 0 & 0 & \gamma & 0 & \theta_{1}^{R} & -\mu -\rho -\theta_{2}^{R} & 0\\ 0 & 0 & 0 & 0 & 0 & \gamma & 0 & \theta_{2}^{R} & -\mu -\rho -\theta_{3}^{R} \end{array} \end{array}\right)$$

$$V_{E_{0}}=\left(\begin{array}{c} \begin{array}{cccc} V_{0} & 0 & 0 & 0\\ 0 & V_{0} & 0 & 0\\ 0 & 0 & V_{0} & 0\\ 0 & 0 & 0 & V_{0} \end{array} \end{array}\right)$$

Step 3:

Obtain basic reproduction number ${}_{M}R_{0}\left(IV\right)=\rho \left(F_{E_{0}}V_{E_{0}}^{-1}\right)$, where ρ represents the maximum eigenvalue.

### Appendix A.3. Initial Population

According to the statistical results of all groups of people in Anhui in 2015, the initial value is shown in Table A1.

**Table A1:** The initial population in 2015.

	Population	Heterosexual Women	Heterosexual Men	Homosexual Men	Bisexual Men
Stage					
*S*		$S^{w}=$ 24,347,402	$S^{mw}=$ 24,682,596	$S^{mm}=$ 449,877	$S^{b}=$ 330,210
$I_{1}$		$I_{1}^{w}=1200$	$I_{1}^{mw}=1216$	$I_{1}^{mm}=1857$	$I_{1}^{b}=1365$
$T_{1}$		$T_{1}^{w}=0$	$T_{1}^{mw}=0$	$T_{1}^{mm}=0$	$T_{1}^{b}=0$
$R_{1}$		$R_{1}^{w}=5$	$R_{1}^{mw}=5$	$R_{1}^{mm}=5$	$R_{1}^{b}=5$
$I_{2}$		$I_{2}^{w}=335$	$I_{2}^{mw}=340$	$I_{2}^{mm}=527$	$I_{2}^{b}=389$
$T_{2}$		$T_{2}^{w}=810$	$T_{2}^{mw}=820$	$T_{2}^{mm}=1240$	$T_{2}^{b}=925$
$R_{2}$		$R_{2}^{w}=60$	$R_{2}^{mw}=61$	$R_{2}^{mm}=85$	$R_{2}^{b}=67$
$I_{3}$		$I_{3}^{w}=335$	$I_{3}^{mw}=340$	$I_{3}^{mm}=527$	$I_{3}^{b}=389$
$T_{3}$		$T_{3}^{w}=810$	$T_{3}^{mw}=820$	$T_{3}^{mm}=1240$	$T_{3}^{b}=925$
$R_{3}$		$R_{3}^{w}=60$	$R_{3}^{mw}=61$	$R_{3}^{mm}=85$	$R_{3}^{b}=67$

### Appendix A.4. Fitted Parameters

Details of the values and references can be found in Table A2,Table A3 and Table A4. The details of other fitting parameters are shown in Table A5. The fitted curves of the two effective reproduction numbers ${}_{G}R_{e}\left(t\right)$ and ${}_{M}R_{e}\left(t\right)$ for four historical periods I, II, III and IV are shown in Figure A1.

**Table A2:** Infection rate: β.

Parameters	Description	Value (95% CI)	Source
$\beta_{1}^{I}$	infection rate of wild strain carriers in stage 1	0.0428 (0.0160, 0.6590)	[15,37]
$\beta_{2}^{I}$	infection rate of wild strain carriers in stage 2	0.0500 (0.0200, 0.0700)	[37]
$\beta_{3}^{I}$	infection rate of wild strain carriers in stage 3	0.1000 (0.0500, 0.1400)	[37]
$\beta_{1}^{T}$	infection rate of wild strain carriers after HAART in stage 1	0.0282 (0.0106, 0.4349)	[14]
$\beta_{2}^{T}$	infection rate of wild strain carriers after HAART in stage 2	0.0330 (0.0132, 0.0462)	[14]
$\beta_{3}^{T}$	infection rate of wild strain carriers after HAART in stage 3	0.0120 (0.0060, 0.0168)	[14,38]
$\beta_{1}^{R}$	infection rate of drug-resistant carriers in stage 1	$\epsilon \beta_{1}^{I}$	
$\beta_{2}^{R}$	infection rate of drug-resistant carriers in stage 2	$\epsilon \beta_{2}^{I}$	
$\beta_{3}^{R}$	infection rate of drug-resistant carriers in stage 3	$\epsilon \beta_{3}^{I}$	
ϵ	transmissibility ratio of drug-resistant to untreated wild strain carriers	fit	

**Table A3:** Disease progression rate: θ.

Parameters	Description	Value: Years	Source
$1/\theta_{1}^{I}$	course of wild strain carriers in stage 1	4	[14]
$1/\theta_{2}^{I}$	course of wild strain carriers in stage 2	6	[14]
$1/\theta_{3}^{I}$	course of wild strain carriers in stage 3	2	[14]
$1/\theta_{1}^{R}$	course of drug-resistant carriers in stage 1	4	[14,39,40]
$1/\theta_{2}^{R}$	course of drug-resistant carriers in stage 2	6	[14,39,40]
$1/\theta_{3}^{R}$	course of drug-resistant carriers in stage 3	2	[14,39,40]

**Table A4:** Other parameters.

Parameters	Description	Value	Source
η	the percentage of patients who give up HAART	0.0602	[41,42]
γ	rate of drug resistance after HAART	0.0545	[42,43]
ρ	the percentage of patients who are no longer resistant	0.2687	[42,44]
μ	natural mortality rate	0.0131	[45]
*k*	ratio of female to other and male to other transmission	0.5263	[36]

**Table A5:** Other fitted parameters.

Period	Parameter	Value/Function
I	$a^{w}$	509,832
	$a^{mw}\left(t\right)$	542,781 − $a^{mm}\left(t\right)$ − $a^{b}\left(t\right)$
	$a^{mm}\left(t\right)$	1432.6 × *t* + 10,527
	$a^{b}\left(t\right)$	1607 × *t* + 10,431
	$c^{mm}\left(t\right)$	−0.036928 × *t* + 2.8484
	$c^{b}\left(t\right)$	−0.040123 × *t* + 3.1024
	$c^{w}$	1.5228
	$c^{mw}$	1.5659
	*v*	0.70368
II	$a^{w}$	613,031
	$a^{mw}\left(t\right)$	685,421 − $a^{mm}\left(t\right)$ − $a^{b}\left(t\right)$
	$a^{mm}\left(t\right)$	1622.4 × *t* + 12,382
	$a^{b}\left(t\right)$	1398.7 × *t* + 10,591
	$c^{mm}\left(t\right)$	−0.042873 × *t* + 3.4122
	$c^{b}\left(t\right)$	0.092656 × *t* + 3.4909
	$c^{w}$	1.4985
	$c^{mw}$	1.5066
	v(t)	−0.0098732 × *t* + 0.59836
	$\delta_{3}\left(t\right)$	0.03733 × *t* + 0.35124
III	$a^{w}$	449,161
	$a^{mw}\left(t\right)$	493,770 − $a^{mm}\left(t\right)$ − $a^{b}\left(t\right)$
	$a^{mm}\left(t\right)$	1455.8 × *t* + 5545.3
	$a^{b}\left(t\right)$	2079.9 × *t* + 5093.9
	$c^{mm}\left(t\right)$	1/(0.61892 + exp(−2.6515 × *t* + 3.1585)) + 3.5015
	$c^{b}\left(t\right)$	1/(0.54199 + exp(−2.7309 × *t* + 3.2935)) + 3.4814
	$c^{w}$	1.5362
	$c^{mw}$	1.5631
	v(t)	0.07004 × *t* + 0.51912
	$\delta_{2}\left(t\right)$	1/(1.6826 + exp(−1.809 × *t* + 0.53915))+ 0.2192
	$\delta_{3}\left(t\right)$	1/(1.6585 + exp(−1.8287 × *t* + 0.55639)) + 0.19956

### Appendix A.5. Sensitivity Analysis on Therapeutic Effect

There are two parameters related to the therapeutic effect: the post-treatment transmission rate ($\beta^{T}$) and the course of disease ($1/\theta^{T}$). Next, we will discuss the effect of changes in these parameters on the effective reproduction number ${}_{M}R_{0}\left(IV\right)$. We keep other parameters unchanged during this process. The sensitivity analysis results are shown in Figure A2b.

If only changing the infection rate after treatment at each infection stage, that is, reducing or increasing $\beta^{T}$, the result is shown by the solid line in the Figure A2b. The abscissa is the multiple of the infection rate after the current baseline treatment (indicated by *p*, that is, the infection rate $\beta^{T}$ becomes $p*\beta^{T}$ after each stage of treatment), and the ordinate is the corresponding ${}_{M}R_{0}\left(IV\right)$ value. The result shows that under the condition that other parameters remain unchanged, only when the infection rate of each infection stage after treatment simultaneously reduces to about 10% of the baseline infection rate after treatment $\beta^{T}$ can ${}_{M}R_{0}\left(IV\right)$ drop below 1 (cyan solid line). Moreover, among the three infection stages, the infection rate in the second stage is the most sensitive to ${}_{M}R_{0}\left(IV\right)$ (solid green line), while the infection rate after treatment in the third stage is the least sensitive to ${}_{M}R_{0}\left(IV\right)$ (solid red line). This is because the course of the second stage is the longest among the three stages, and the patients in the third stage of infection have weaker sexual ability.

If only changing the course of the disease after treatment at each infection stage ($1/\theta^{T}$), the result is shown as the dotted line in Figure A2b, where the abscissa is *p*, and it is the value of a multiple of the course parameter value after current baseline treatment. We are surprised to find that under the condition that other parameters remain unchanged, ${}_{M}R_{0}\left(IV\right)$ is positively correlated with the course of disease after treatment, that is, the longer the course of disease after treatment, the higher the ${}_{M}R_{0}\left(IV\right)$ will be. Moreover, only when the course of the disease after each stage of treatment is reduced to less than 25% of its baseline parameter value at the same time can ${}_{M}R_{0}\left(IV\right)$ drop below 1 (cyan dotted line). The order of sensitivity to ${}_{M}R_{0}\left(IV\right)$ in the course of the disease after treatment at different infection stages is consistent with the sensitivity to drug infectivity.

### Appendix A.6. Sensitivity Analysis on the Number of Sexual Partners

If other parameters remain unchanged, and only the number of annual sexual partners of various groups of people is changed, the result is shown in Figure A3a, where the abscissa is the multiple of the current parameter change, and the ordinate is the corresponding ${}_{M}R_{0}\left(IV\right)$ value. The greater the slope of the straight line, the greater the influence of this parameter is on ${}_{M}R_{0}\left(IV\right)$. The results show that the disease transmission can be effectively controlled when the annual number of sexual partners in all populations is reduced to 0.4 times the current number of sexual partners (cyan line). The order of the influence of the number of different groups annual sexual partners on ${}_{M}R_{0}\left(IV\right)$ is: homosexual male (green line) > bisexual male (red line) > heterosexual people (the blue line, see the illustration on the Figure A3a). An interesting fact is that changing the number of heterosexual people’s sexual partners has little impact on the spread of HIV/AIDS (blue line): almost negligible. The two types of people with homosexual sexual behavior (green line and red line) contributed a significant effect.

We further analyzed two types of MSM population and explored the impact of the number of sexual partners of bisexual men ($c^{b}$) and the number of heterosexual men ($c^{mm}$) on ${}_{M}R_{0}\left(IV\right)$ (Figure A3b).

### Appendix A.7. Influence of Bisexual Men’s Sexual Preference on HIV/AIDS Prevalence

Traditionally, we believe that bisexual men play a bridge role in HIV transmission because they are not only involved in the transmission between homosexual groups but also in the transmission between heterosexual people. We would like to know what impact bisexual men would have on the HIV epidemic if they become purely homosexual or heterosexual. Figure A4a is the curve of the number of HIV-positive patients in the whole population of Anhui province from 2020 to 2025 over time after changing the nature of bisexual males. The results show that under the assumption that bisexual men are the pure MSM population, the total number of HIV/AIDS infections in Anhui province has shown a rapid upward trend, far exceeding the baseline status, and the number of surviving infections will increase to 38,171 by 2025 (95% CI: 34,694–42,078, the red curve in Figure A4a), and the number of new infections will also increase to 6333 accordingly. However, the resistance rate is lower than the baseline status and will decrease to 9.41% by 2025 (95% CI: 8.83–9.88%, the red line in Figure A4b). On the contrary, if you disconnect from male sex and become purely heterosexual, the number of HIV infections will be significantly lower than the baseline status by 2025, it will be as low as 26,957 by 2025 (95% CI: 25,890–28,633, Figure A4a green curve), and the number of new infections will reduce to 2465. Meanwhile, its resistance rate will exceed the baseline status, increasing to 10.59% (95% CI: 10.14–10.93%) (the green curve in Figure A4b) in 2025.

### Appendix A.8. Prevention and Control Strategy Optimization Analysis

For the current prevention and control measures, the spread of HIV in Anhui province has not been well controlled. Three important measures are now considered to be strengthened, and the new infection rate and the rate of drug resistance in 2021–2025 are numerically simulated (see the results in Table A6 and Table A7).

The measures of reinforcement are to increase the treatment rate, reduce the withdrawal rate, and reduce the number of sexual partners. The specific measures are as follows:

(1). The treatment rate is increased by 10%;
(2). The treatment rate is increased by 20%;
(3). The withdrawal rate is reduced by 10%;
(4). The withdrawal rate is reduced by 20%;
(5). The number of sexual partners is reduced by 10%;
(6). The number of sexual partners is reduced by 20%;
(7). (1)(3)(5) combination;
(8). (2)(4)(6) combination.

From Table A6 and Table A7, it is not difficult to see that increasing prevention and control efforts can reduce the population of new infections, and at the same time, it will also reduce the primary drug resistance population. The greater the prevention and control efforts are, the more the new infections and primary drug resistance population reduce. Among the three measures, the best effect is to reduce the number of sexual partners. Combining the three measures (cases 7, 8) compared with one measure is more effective in controlling both new infections and primary drug resistance population. For the prevention and control of HIV in Anhui, China, comprehensive measures can not only control new infections but also reduce the population of primary drug resistance, thus being a wise choice.

**Table A6:** New infection population under different measures.

	Years	2021	2022	2023	2024	2025
Measures						
Baseline parameter (95% CI)		3194	3499	3831	4207	4619
		(2829, 3560)	(3031, 3970)	(3257, 4434)	(3511, 4955)	(3780, 5541)
(1) The treatment rate is increased by 10% (95% CI)		3150	3432	3752	4102	4497
		(2787, 3510)	(2981, 3884)	(3193, 4325)	(3424, 4820)	(3670, 5378)
(2) The treatment rate is increased by 20% (95% CI)		3007	3258	3554	3875	4234
		(2678, 3360)	(2855, 3714)	(3049, 4122)	(3269, 4585)	(3502, 5110)
(3) The withdrawal rate is reduced by 10% (95% CI)		3128	3416	3738	4094	4495
		(2787, 3500)	(2982, 3893)	(3201, 4346)	(3450, 4861)	(3708, 5437)
(4) The withdrawal rate is reduced by 20% (95% CI)		3069	3336	3656	4002	4392
		(2740, 3448)	(2983, 3937)	(3143, 4279)	(3381, 4781)	(3628, 5344)
(5) The number of sexual partners is reduced by 10% (95% CI)		2781	2991	3223	3474	3755
		(2474, 3113)	(2613, 3406)	(2768, 3740)	(2940, 4106)	(3123, 4502)
(6) The number of sexual partners is reduced by 20% (95% CI)		2326	2456	2602	2757	2925
		(2062, 2601)	(2142, 2788)	(2232, 2999)	(2332, 3228)	(2435, 3477)
(1)(3)(5) combination (95% CI)		2780	2927	3153	3401	3677
		(2502, 3145)	(2608, 3424)	(2766, 2744)	(2931, 4094)	(3104, 4494)
(2)(4)(6) combination (95% CI)		2309	2416	2540	2680	2831
		(2056, 2583)	(2119, 2743)	(2200, 2929)	(2291, 3138)	(2384, 3373)

**Table A7:** Primary drug resistance population under different measures.

	Years	2021	2022	2023	2024	2025
Measures						
Baseline parameter (95% CI)		209	235	262	292	323
		(188, 230)	(209, 261)	(231, 294)	(253, 331)	(276, 371)
(1) The treatment rate is increased by 10% (95% CI)		208	234	261	291	321
		(187, 229)	(209, 260)	(231, 295)	(255, 333)	(277, 374)
(2) The treatment rate is increased by 20% (95% CI)		201	228	257	287	318
		(181, 222)	(204, 255)	(227, 189)	(251, 327)	(274, 368)
(3) The withdrawal rate is reduced by 10% (95% CI)		206	231	258	287	318
		(185, 227)	(207, 258)	(229, 291)	(251, 327)	(274, 367)
(4) The withdrawal rate is reduced by 20% (95% CI)		202	228	254	283	313
		(183, 224)	(205, 255)	(226, 288)	(249, 325)	(272, 365)
(5) The number of sexual partners is reduced by 10% (95% CI)		184	205	227	250	273
		(168, 199)	(183, 228)	(201, 256)	(218, 284)	(236, 315)
(6) The number of sexual partners is reduced by 20% (95% CI)		155	172	188	205	221
		(139, 172)	(153, 192)	(166, 212)	(179, 232)	(190, 254)
(1)(3)(5) combination (95% CI)		176	197	219	241	264
		(157, 194)	(175, 219)	(192, 246)	(209, 275)	(226, 305)
(2)(4)(6) combination (95% CI)		154	170	184	201	219
		(137, 174)	(151, 190)	(164, 210)	(178, 234)	(189, 250)
